# Support and Adjustments for Neurodiverse Students Referred to Greater Manchester Universities Student Mental Health Service

**DOI:** 10.1192/bjo.2025.10482

**Published:** 2025-06-20

**Authors:** Emmalene Fish, Timothy Alnuamaani, Simon Postlethwaite

**Affiliations:** 1 Pennine Care NHS Foundation Trust, Greater Manchester, United Kingdom; 2 Greater Manchester Mental Health, Greater Manchester, United Kingdom

## Abstract

**Aims:** Are neurodiverse university students, referred to Greater Manchester University Student Mental Health Service (GMUSMHS), receiving the Disability and advisory support services (DASS) input they are entitled to? If not, are GMUSMHS recognising this and signposting appropriately?

**Methods:** In the interest of improving equality and diversity, along with access to higher education, services to support the increasing numbers of neurodiverse students are available. GMUSMHS uses a needs-led approach, across the 5 Greater Manchester universities. It can also ensure those struggling with complex mental health needs, alongside neurodivergence, can be signposted to appropriate educational support/adjustments. Enabling them to thrive and reach their academic potential.

16 referrals mentioning Neurodiversity within the designated 3-month period were identified. Initial referral forms, GMUSMHS assessment notes and outcomes were reviewed for DASS input. There were no exclusion criteria.

**Results:** From our sample 69% had a working diagnosis of Autistic spectrum condition (ASC). In all cases, where the diagnosis was not confirmed, the student was offered screening or onward referral for diagnosis. Six of the students were not noted to be under DASS and were not documented to have been signposted. All students who engaged with GMUSMHS were offered an intervention, this included extended assessment, case management, formulation sessions, Connecting people, emotion regulation/compassion focused therapy group, and PTSD/trauma education group.

**Conclusion:** Neurodivergent students may not be accessing the educational support and adaptations they are entitled to. GMUSMHS are supporting referrals for diagnosis of neurodiversity. They are not, however, consistently documenting if these students are accessing, or have been referred to DASS. Raising awareness and improving a collaborative approach between GMUSMHS and DASS is required. Including prompts on referral forms and assessment proformas may be helpful. A neurodivergent service lead has been allocated and service development to improve access for this client group has begun.

